# Early Detection of Cerebral Infarction With Middle Cerebral Artery Occlusion With Functional Near-Infrared Spectroscopy: A Pilot Study

**DOI:** 10.3389/fneur.2018.00898

**Published:** 2018-11-08

**Authors:** Hyuksool Kwon, Kyuseok Kim, You Hwan Jo, Min Ji Park, Sang-Bae Ko, Tae Jung Kim, Jihoon Kang, Hyeon-Min Bae, Ji Eun Lee

**Affiliations:** ^1^Department of Emergency Medicine, Seoul National University Bundang Hospital, Seongnam-si, South Korea; ^2^Department of Emergency Medicine, Seoul National University College of Medicine, Seoul, South Korea; ^3^Department of Emergency Medicine, Mediplex Sejong Hospital, Incheon, South Korea; ^4^Department of Neurology, Seoul National University Hospital, Seoul, South Korea; ^5^Department of Neurology, Seoul National University Bundang Hospital, Sungnam-si, South Korea; ^6^Department of Electrical Engineering, Korea Advanced Institute of Science and Technology, Daejeon, South Korea

**Keywords:** cerebral infarction, perfusion, middle cerebral artery, computed tomography, magnetic resonance imaging

## Abstract

**Background:** NIRSIT, a functional near-infrared spectroscopy (fNIRS) device with 204 channels, can measure oxyhemoglobin (HbO2) and deoxyhemoglobin (HbR) in non-pulsatile blood flow non-invasively using the absorption difference between HbO2 and HbR at a wavelength of 700–1,000 nm and can display the perfusion status in real time.

**Objective:** We applied NIRSIT to patients with stroke to evaluate the usefulness of NIRSIT as an fNIRS device for the early detection of stroke.

**Methods:** We performed a prospective pilot study in an emergency department (ED). Adult patients who had suspected symptoms and signs of stroke within 12 h of the first abnormal time and who underwent intravenous thrombolysis (IVT) or intra-arterial thrombectomy with acute middle cerebral artery (MCA) or internal carotid artery (ICA) infarction were enrolled. NIRSIT was applied to the patients before the imaging study, and the perfusion status of the brain was displayed in real time at the bedside. We compared the NIRSIT results with the mean transit time (MTT) map from perfusion computed tomography (PCT) and the time-to-peak (TTP) map from perfusion-weighted magnetic resonance imaging (PWI).

**Results:** Six male and three female patients were enrolled, and the median age was 74 years. The most common symptom was unilateral extremity weakness (77.8%), followed by dysarthria (33.3%) and aphasia (11.1%). The median National Institutes of Health Stroke Scale (NIHSS) score was 17. All cases of MCA infarction showed different cerebral oxygen saturation values between bilateral lobes of the brain in fNIRS imaging, and these values matched the PCT and PWI results.

**Conclusions:** The brain hemisphere with low oxygen saturation on fNIRS showed hypoperfusion on PCT or PWI. The fNIRS device could be useful in assessing the perfusion status of the brain and detecting MCA or ICA infarction in real time at the bedside.

## Introduction

Patients with ischemic stroke in the middle cerebral artery (MCA) or internal carotid artery (ICA) region exhibit high rates of mortality and disability, and nearly half of survivors never regain functional independence (1). Rapid assessment of perfusion status with a prompt diagnosis would allow timely treatment with updated and efficient recanalization therapy to ensure a good outcome. Recently developed perfusion scans, such as perfusion computed tomography (PCT) and perfusion-weighted magnetic resonance imaging (PWI), would help in the selection of values to delineate perfusion status. These modalities determine the need for early and rapid interventions, including intravenous thrombolysis (IVT) and intra-arterial thrombectomy, to preserve neurological function (2). However, to obtain these images, it is necessary to move the patients to a specially equipped room.

Recent studies have shown that stroke patients with large vessel occlusions who are treated with targeted endovascular thrombectomy demonstrate a two-fold improvement in the modified Rankin Score post stroke (3). This result suggests that improved access to interventional stroke care may yield improved overall patient outcomes. The American Heart Association has developed a severity-based stroke triage algorithm for the pre-hospital emergency medical service system that highlights the need for the identification of large vessel occlusions, followed by the transport of patients to facilities where interventions may be performed (4). In this context, the pre-hospital recognition of large vessel occlusions could determine the most appropriate hospital destination for such patients because endovascular interventions are available only at facilities with trained interventional teams (5). However, the most accurate tool for evaluating large vessel occlusions is unclear. Thus, there is a need for real-time assessment of cerebral perfusion at the pre-hospital stage and for emergency department (ED) monitoring during the peri-intervention period.

Functional near-infrared spectroscopy (fNIRS) is an emerging technique that allows non-invasive monitoring of the perfusion status in multiple areas, such as the carotid artery during cardiac surgery or in patients with atherosclerotic occlusive diseases (6–11). This technique facilitates effective real-time measurement of cerebral perfusion. Near-infrared spectral tomography (NIRSIT) (OBELAB Inc., Kangnam-gu, Seoul, Korea) was developed as a brain imaging system, and this portable, wireless and wearable fNIRS device can assess perfusion status at the bedside in real time (6). NIRSIT measures oxyhemoglobin (HbO2), deoxyhemoglobin (HbR), and regional oxygen saturation (rSO2) with improved spatial resolution using the difference in the absorption rate of near-infrared light.

This study was conducted to evaluate the usefulness and accuracy of NIRSIT in the evaluation of patients in the acute stage of cerebral infarction with MCA occlusion in the ED in comparison to the conventional mean transit time (MTT) map from PCT and the time-to-peak (TTP) map from PWI.

## Methods

### Study design and setting

We conducted a prospective, observational pilot study that compared fNIRS to conventional PCT and PWI in an urban, tertiary care ED from July 2016 to December 2016. The study was approved by the Institutional Review Board of Seoul National University Bundang Hospital, and the patients or their caregivers were informed about the study and gave written consent.

### Selection of participants

**Figure 1:**
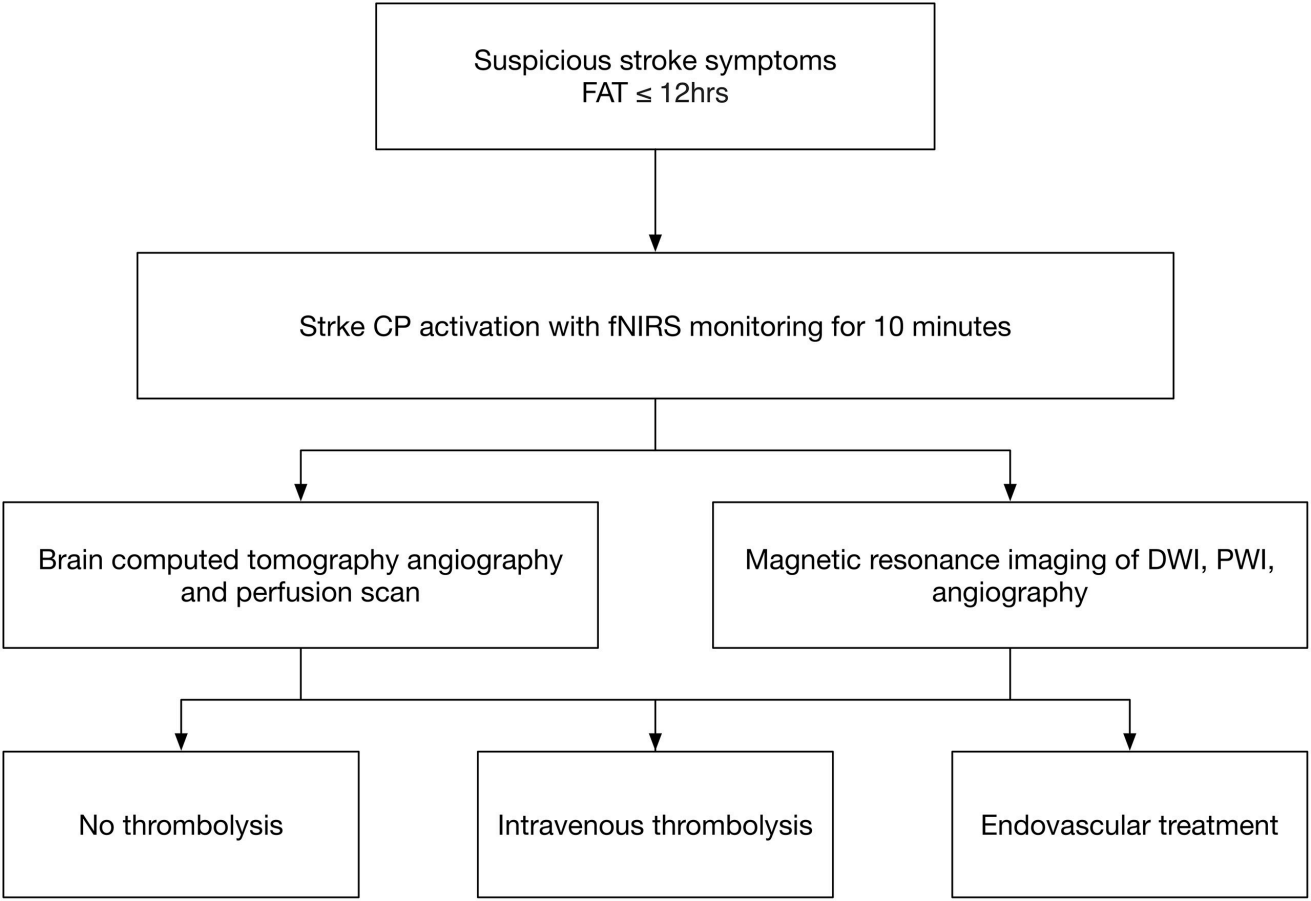
Stroke critical pathway. FAT indicates the first abnormal time, which is the first notification time by patients or witnesses; CP indicates the critical pathway for stroke; IV thrombolysis indicates intravenous thrombolysis; and EVT indicates endovascular treatment.

Patients who had suspected MCA or ICA infarction were enrolled in the ED with stroke critical pathway (CP) activation. The stroke CP is activated in patients who are at least 18 years of age and have suspected signs and symptoms of stroke within 12 h of the first abnormal time (Figure 1) (12). The signs and symptoms of acute stroke are an acute onset of altered consciousness, slurred speech or language disturbance, lateralized sensorimotor loss, visual loss or double vision, hemi-neglect, and ataxia or imbalance.

Patients were excluded if they had symptoms due to trauma or hypoglycemia. Each patient's neurologic impairment was quantified with the National Institutes of Health Stroke Scale (NIHSS). Stroke mechanisms were classified into five subtypes according to the modified Trial of ORG 10172 in Acute Stroke Treatment (TOAST) classification system (13).

### Device and measures

**Figure 2:**
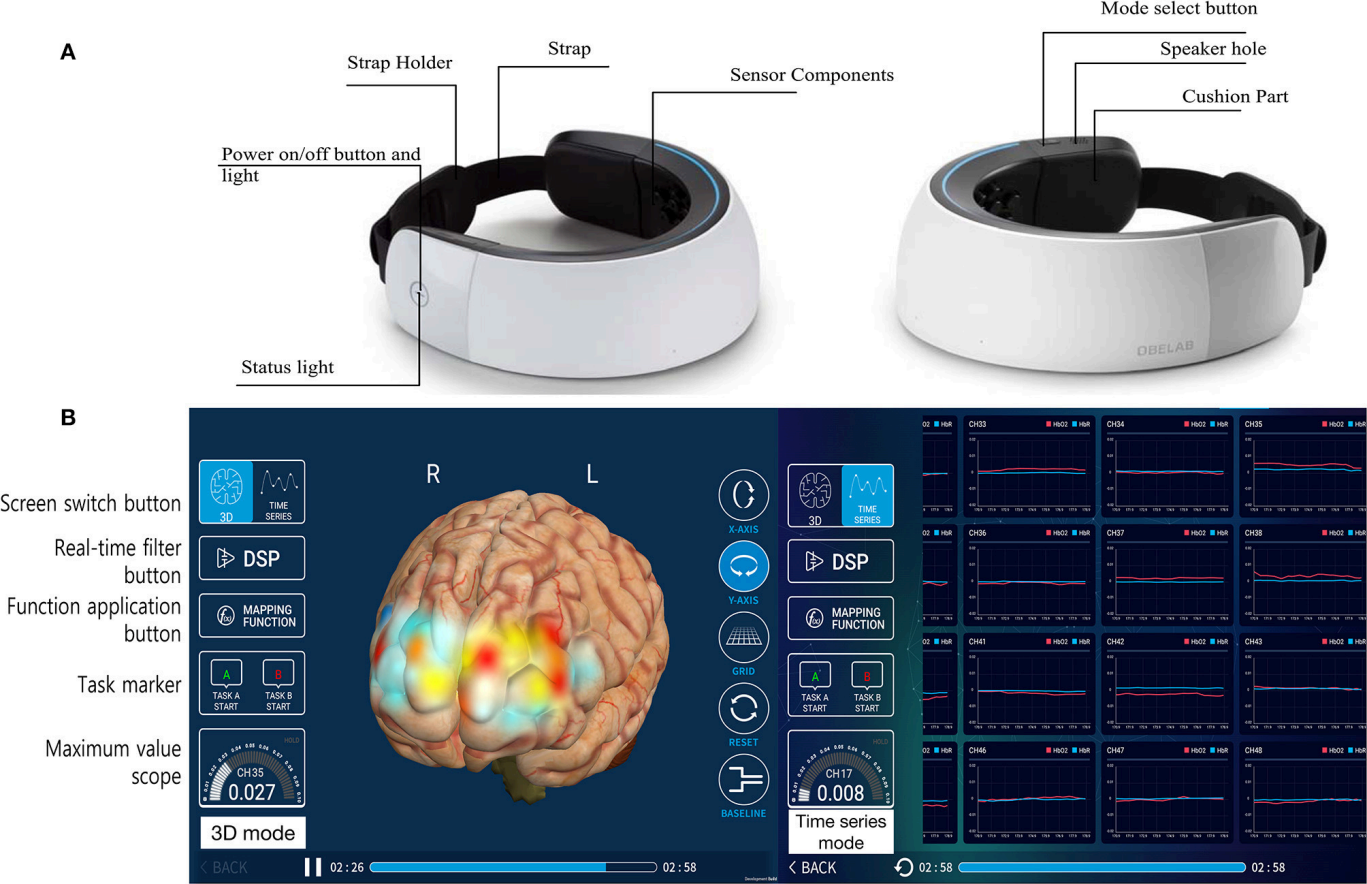
NIRSIT (a wearable fNIRS device with 204 channels). The NIRSIT device comprised the measurement sensor components (white outer surface area) and the strap portion **(A)**. The NIRSIT software is operated wirelessly on mobile tablets that present the real-time status using monitoring applications **(B)**. The measured data directly visualize the change in hemoglobin level as a color map on a 3-dimensional hemisphere image (left side of figure), and the actual values of oxygenated hemoglobin are displayed in a time series graph (right side of figure).

NIRSIT is a wearable fNIRS device that measures HbO2, HbR, and rSO2 using the difference in the absorption rate of near-infrared light through the cerebral cortex. The light is < 5 mW in power, so the use of the device is harmless to the user. NIRSIT uses 204 channels attached to the forehead of the patient for measurements. Four by 51 channels are located in the frontotemporal area of a brain (Supplementary Figure 1, also available at https://fccid.io/2AHYINIRSIT/User-Manual/User-Manual-3032004). Measurements are visualized using an application on a tablet PC with diffuse optical tomography (Galaxy Tab, Samsung, Seoul, Korea) (Figure 2). Diffuse optical tomography estimates the spatial distribution of absorption characteristics of a brain in 3D structure with fine spatial resolution.

NIRSIT uses a continuous wave to obtain a regional hemodynamic response from the prefrontal lobe. This device weighs 450 g, making it light and suitable for use as a portable device. NIRSIT has two wavelengths, the 780 nm wavelength that is absorbed by HbR and the 850 nm wavelength that is equally absorbed by HbO2 and HbR. rSO2 was calculated as the concentration percentage HbO2/(HbO2 + HbR) × 100 (Supplementary Figure 2). In addition to rSO2 monitoring, NIRSIT allows clinicians to monitor the patients' brain oxygenation in an intuitive way using 3D brain maps. Unlike other NIRS devices that have a spatial resolution of 3 × 3 cm, NIRSIT has improved its spatial resolution to a maximum of 0.4 × 0.4 cm, which is comparable to that of functional magnetic resonance imaging (0.3 × 0.3 cm).

The color shown in 3D brain imaging reflects the degree of oxygenation at each location in the brain. Based on this principle, we compared the 3D brain image obtained with the NIRSIT device to the MTT map obtained with PCT and the TTP map obtained with PWI in patients with MCA or ICA infarction.

### Study protocol and data collection

When the stroke CP was activated by a triage nurse, the system automatically contacted the attending neurologists and the emergency physicians. The engineers in the computed tomography and magnetic resonance imaging rooms were also notified for rapid imaging. After completion of the clinical assessment by history collection and neurological examination, imaging studies were performed, and the treatment plan, including IVT and intra-arterial thrombolysis, was determined. NIRSIT was applied to the patient's forehead by the neurologist during the neurological examination and during the preparation time for the transfer of the patient from the ED to the computed tomography or magnetic resonance imaging room. The patient's position was supine, and NIRSIT was applied for 3–10 min to obtain data about the hemodynamic responses in the cortical regions. The detailed operation manual is available at https://fccid.io/2AHYINIRSIT/User-Manual/User-Manual-3032004.

### Data analysis

The primary objective of this study was to explore the cerebral tissue saturation measured by NIRSIT compared to that measured by the conventional MTT map from PCT or the TTP map from PWI to evaluate the acute stage of cerebral infarction with MCA occlusion. There is no statistical analysis because the data are largely presented as a series of nine case studies with visual comparisons to PCT and PWI. Values are expressed as the median and the interquartile range (IQR) or number (%).

## Results

### Patient summaries

A total of nine patients were enrolled in the study. Among the 9 patients, 6 (66.7%) were male, and the median age was 74 (68.0–82.5) years. The most common symptom was unilateral extremity weakness (77.8%), followed by dysarthria (33.3%) and aphasia (11.1%). The median NIHSS score of the patients was 17 (IQR: 10.5–19.5) (Table 1).

**Table 1:** Characteristics and diagnosis of the study patients.

**No**	**Infarction territory**	**Age (range)**	**Chief complaint**	**NIHSS**	TOAST^*^
1	Left MCA infarction	86–90	R hemiparesis Aphasia	17	UD
2	Left MCA infarction	61–65	R hemiparesis Altered mental status	16	LAA
3	Left ACA/MCA infarction	86–90	R hemiparesis Aphasia	26	OD
4	Right MCA infarction	71–75	L hemiparesis Dysarthria	3	LAA
5	Right MCA infarction	76–80	L hemiparesis Dysarthria	5	CE
6	Right MCA infarction	76–80	L hemiparesis	18	UD
7	Right MCA infarction	51–55	L hemiparesis	18	LAA
8	Left MCA infarction	71–75	R hemiparesis	21	CE
9	Left MCA infarction	71–75	R hemiparesis Dysarthria	16	LAA

* *Trial of ORG 10172 in Acute Stroke Treatment (TOAST) classification system: large-artery atherosclerosis (LAA), cardioembolism (CE), small-artery occlusion, other determined etiology (OD), and undetermined etiology (UD). R, right; L, left; MCA, middle cerebral artery*.

### Comparison of 3D mapping with fNIRS, PCT, and PWI

**Figure 3:**
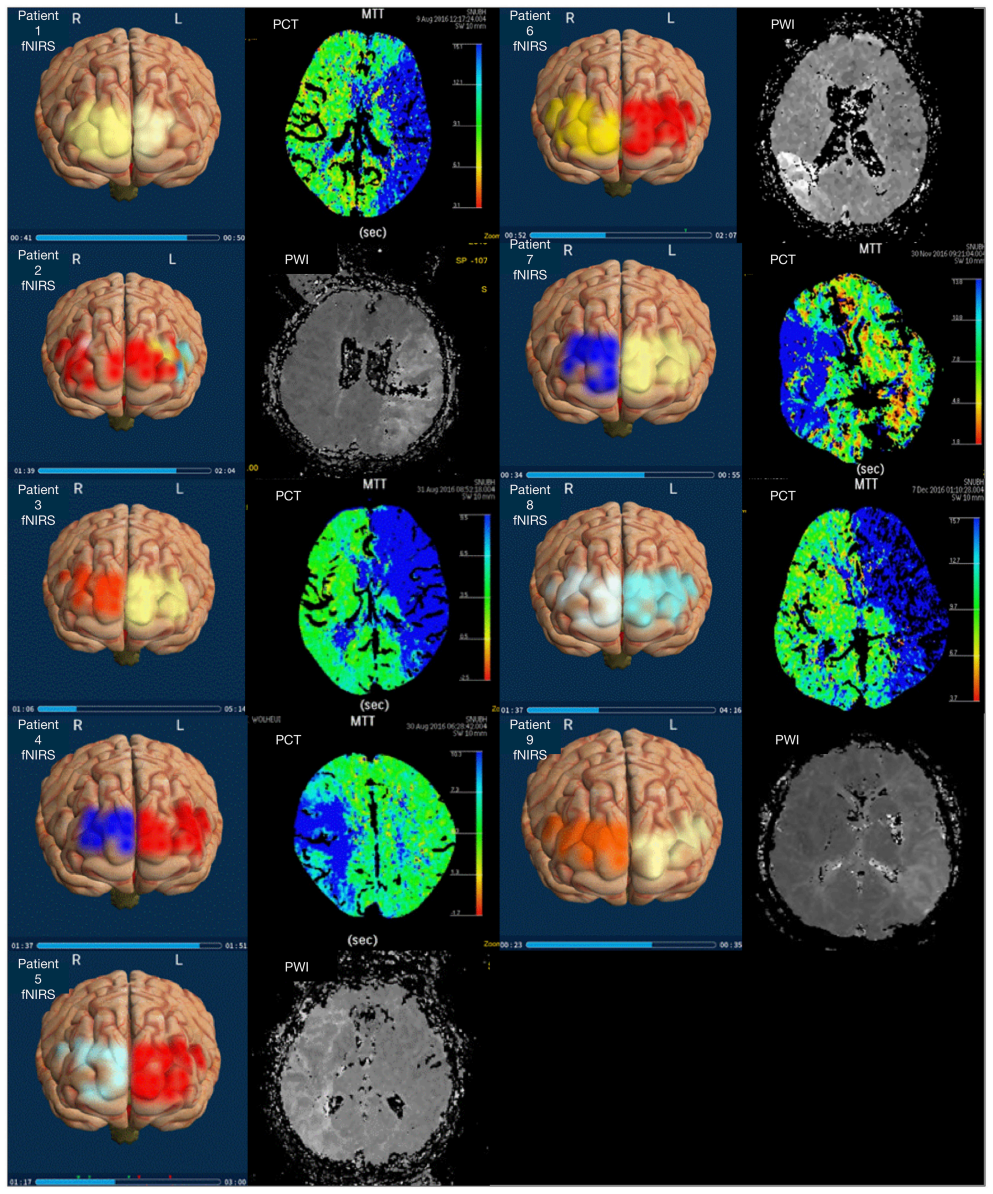
Images from fNIRS vs. PCT or PWI. This figure shows the comparison of fNIRS images and PCT or PWI images from MCA infarction patients. Patient 1 showed poor or brighter yellow color of the left frontal lobe on the 3D display (left side) and a perfusion delay (blue color) in the left MCA territory on the MTT map (right side). Patient 2 showed asymmetric yellow and blue coloration of the left frontal lobe and a perfusion delay in the left MCA territory on the TTP map (white color). Patients 3, 4, 7, and 8 showed a perfusion delay on the MTT map with compatible asymmetric color mapping of the 3D frontal cortex. Patients 5, 6, and 9 a showed perfusion delay on the TTP map with asymmetric color mapping. The TTP map from PWI showed a perfusion delay in the whole left MCA territory with a multifocal, small-diffusion restricted lesion in the left MCA territory on diffusion-weighted imaging (DWI). The average right and left cerebral rSO2 values were 87.15 and 68.25%, respectively. No, number; R, right; L, left; MCA, middle cerebral artery; MTT, mean transit time; sec, second; PCT, perfusion computed tomography; PWI, perfusion-weighted magnetic resonance image.

Representative perfusion PCT, PWI, and NIRSIT images from the patients are shown in Figure 3. All nine cases showed definite asymmetric cerebral oxygen saturation between the two hemispheres in fNIRS imaging. The HbO2 level in the NIRSIT group strongly correlated with hypoperfusion on PCT or PWI.

#### Patient 1

Patient 1 was 86–90 years old with hypertension, diabetes and previous sigmoid colon cancer, and he visited the ED with a 30 min history of right hemiparesis. His NIHSS score was 17. The MTT map from PCT showed a perfusion delay in the whole left MCA territory with occlusion of the left distal portion of the main stem of the middle cerebral artery (M1) on CT angiography (CTA). The average values of the right and left cerebral rSO2 were 63.29 and 60.64%, respectively. He was treated with endovascular treatment (EVT) and reached the complete recanalization state.

#### Patient 2

Patient 2 was 61–65 years old with a history of hypertension and diabetes, and he visited the ED with a 50 min history of altered mental status and right hemiparesis. His NIHSS score was 26. The TTP map from PWI showed a perfusion delay in the whole left MCA territory with a multifocal, small-diffusion restricted lesion in the left MCA region on diffusion-weighted imaging (DWI). The average right and left cerebral rSO2 values were 87.15 and 68.25%, respectively. He was treated with combined recanalization therapy, comprising both IVT and EVT.

#### Patient 3

Patient 3 was 86–90 years old with hypertension, diabetes and previous sigmoid colon cancer. Her caregiver found that her altered mental status remained altered after 5 h 30 min, and she arrived at the hospital via emergency medical service. The MTT map showed a significant perfusion delay of the whole MCA and anterior cerebral artery (ACA) with occlusion of a distal cavernous portion of the ICA. The average right and left cerebral rSO2 values were 63.29 and 60.64%, respectively. The infarction cores from the cerebral blood volume map and MTT map were mismatched. She was treated with EVT.

#### Patient 4

Patient 4 was 71–75 years old with hypertension, and she visited the ED with a 5 h history of left hemiparesis with dysarthria. Her NIHSS score was 3, and the MTT map from PCT showed a perfusion delay in the right MCA territory with severe stenosis in the superior portion of the right minor branch and moderate stenosis in the right main branch. The average right and left cerebral rSO2 values were 69.01 and 71.28%, respectively. She was treated with EVT.

#### Patient 5

Patient 5 was 71–75 years old with hypertension and atrial fibrillation, and he visited the ED with a 250 min history of left hemiparesis and dysarthria. His NIHSS score was 5, and the TTP map showed a significant perfusion delay in the right MCA territory with internal border zone area acute infarctions on DWI. The average right and left cerebral rSO2 values were 61.52 and 65.47%, respectively. He was treated with combined recanalization therapy for occlusion of the proximal ICA.

#### Patient 6

Patient 6 was 76–80 years old with hypertension and diabetes, and she visited the ED with a 125 min history of left hemiparesis. Her NIHSS score was 18, and the TTP map from PWI showed a perfusion delay in the inferior division region of the right MCA and a mild perfusion delay in the other MCA territory. The average right and left cerebral rSO2 values were 60.64 and 63.29%, respectively. She was treated with combined treatment for occlusion of the proximal portion of M1.

#### Patient 7

Patient 7 was 51–55 years old without underlying disease, and he visited the ED with a 45 min history of left hemiparesis. His NIHSS score was 18, and the MTT map showed a perfusion delay in the whole right MCA territory. The average right and left cerebral rSO2 values were 48.35 and 64.87%, respectively. He was treated with EVT.

#### Patient 8

Patient 8 was 71–75 years old with hypertension, diabetes and atrial fibrillation, and he visited the ED with a 44 min history of right hemiparesis and stupor mentality. His NIHSS score was 21, and the MTT map from PCT showed a perfusion delay with a large MTT/cerebral blood volume mismatch in the left MCA and ACA territories. The average right and left cerebral rSO2 values were 71.01 and 39.36%, respectively. He was treated with combined recanalization therapy for occlusion of the proximal ICA.

#### Patient 9

Patient 9 was 71–75 years old with hypertension, and he visited the ED with a 102 min history of right hemiparesis and dysarthria. His NIHSS score was 16, and the TTP map showed a mild perfusion delay in the whole MCA territory with a perfusion defect area at the basal ganglia and corona radiata. The average right and left cerebral rSO2 values were 67.32 and 61.26%, respectively. He was treated with EVT, and the final diagnosis was left MCA infarction with left main branch occlusion.

## Discussion

We presented the first clinical study on the feasibility of an fNIRS device (NIRSIT) as a portable device to aid in the early detection of MCA or ICA infarction. In all nine cases, NIRSIT detected all MCA infarctions. NIRSIT could be applied in a very short time, and the researchers could intuitively interpret the results by the colored display of perfusion in 3D brain imaging. These findings support the most beneficial aspect of NIRSIT, which has the ability to record and display the perfusion status immediately in partial or whole cortical regions in real time at the bedside.

fNIRS is an emerging non-invasive, non-ionizing, and relatively low-cost neuroimaging technique that uses the ability of light in the near-infrared spectrum (700–1,000 nm) to penetrate biological tissue to assess changes in HbO2, HbR, blood volume, and tissue oxygen availability with high temporal resolution (14). Based on the principle that the absorption coefficients of HbO2 and HbR are different in the near-infrared region, fNIRS can extract the perfusion status of the brain in real time by measuring the intensity variation in the near-infrared light that has traveled through the tissues. This capability allows for the assessment of rSO2 changes by a comparison of the HbO2 and HbR concentrations. Based on these principles, several studies have used fNIRS in different ways to monitor and evaluate patients with cerebral infarction and determine severity (6–11, 14–17). In these studies, cerebral ischemia was characterized mainly by a marked decrease in HbO2 and an increase in HbR rather than a mild increase in cerebral blood volume. Although there was no large-scale study, these findings suggested that acute decreased perfusion and ischemia could be easily monitored at the bedside with the fNIRS device.

It is not always easy to identify patients with cerebral infarction correctly by symptoms alone. In addition, stroke mimics are caused by a variety of diseases, and they can delay accurate diagnosis, especially in pre-hospital settings. The critical step in the early differentiation of stroke from stroke mimics is rapid brain imaging. Previous studies reported that rapid notification of emergency medical services using a stroke code system for rapid brain imaging reduces the door-to-computed tomography time before hospital arrival (18). fNIRS may have advantages in the early detection of stroke, but no study has been performed in the pre-hospital setting for effective real-time measurement of cerebral perfusion. We performed this study to evaluate the usefulness of fNIRS immediately after patient arrival at the emergency room to mimic the pre-hospital setting.

NIRSIT has several advantages for the early assessment of perfusion status and the diagnosis of stroke. First, rSO2 is expressed in real time and in color, making it easy to detect a cerebral perfusion delay intuitively. Second, there is no concern about radiation exposure or a shielding facility, making NIRSIT superior in terms of cost-effectiveness. Finally, in some cases, NIRSIT might help explain the patient's local perfusion status. Patients 6 and 9 showed a mild perfusion delay status rather than clinical symptoms or vascular status, and NIRSIT provided additional evidence of asymmetric rSO2 values between the hemispheres.

There are several limitations of this study. First, the sample size was small because this was a pilot study of the application of NIRSIT in ischemic stroke patients. We are planning a larger clinical study. Second, this device was attached to the forehead and detected the rSO2 of the area. All nine cases had large infarction sizes. The measurement did not reflect the lacunar part of the infarction in the basal ganglia or the posterior circulation of the brain. Third, we did not present cerebral hemorrhage. In the cases of subdural hematoma, no signal was detected at the site of bleeding, and further study is necessary. Fourth, we could not compare the measurements acquired from fNIRS and PCT or PWI because the sensors of NIRSIT cover the frontotemporal area, not the whole brain. In addition, we focused on a visual comparison for early detection of cerebral infarction. Fifth, we did not apply NIRSIT after thrombolysis or thrombectomy, and we could not compare the changes during these procedures. To further ensure the clinical usefulness and efficacy of NIRSIT, further studies are necessary with large numbers of patients in the ED and at the pre-hospital stage.

## Conclusion

NIRSIT (an fNIRS device with 204 channels) might help with the early recognition of stroke without the use of radiation or contrast agents by reflecting differences in the oxygen saturation levels in the bilateral frontotemporal lobes. NIRSIT may be easily applied in the ED and at the pre-hospital stage, thus facilitating the early detection of stroke, the transfer of patients to the appropriate institution, and early intervention.

## Data availability statement

Datasets are available on request. The raw data supporting the conclusions of this manuscript will be made available by the authors, without undue reservation, to any qualified researcher.

## Ethics statement

After a full description of the study, all subjects gave written informed consent in accordance with the Declaration of Helsinki. The protocol was approved by the local institutional review board at Seoul National University Bundang Hospital (local reference number B-1607-354-406).

## Author contributions

HK and KK contributed equally to the conceptualization and design of the study, and they also drafted the initial manuscript, and approved the final manuscript as submitted. YJ coordinated and supervised data collection, critically reviewed the manuscript, and approved the final manuscript as submitted. MP, S-BK, TK, and JK carried out the initial analyses and reviewed, and revised the manuscript. H-MB developed the instrument. JL collected all clinical data. All authors approved the final manuscript as submitted and agree to be accountable for all aspects of the work.

### Conflict of interest statement

The authors declare that the research was conducted in the absence of any commercial or financial relationships that could be construed as a potential conflict of interest.
