# Supplementary material for: Early Detection of Cerebral Infarction With Middle Cerebral Artery Occlusion With Functional Near-Infrared Spectroscopy: A Pilot Study
Source: Front Neurol. 2018 Nov 8;9:898. doi: 10.3389/fneur.2018.00898 (PMC6236112; doi:10.3389/fneur.2018.00898)

Supplementary Material

Early detection of cerebral infarction with middle cerebral artery occlusion with functional near-infrared spectroscopy: a pilot study

Hyuksool Kwon, MD*, Kyuseok Kim, MD, PhD, You Hwan Jo, MD, PhD, Min Ji Park, MD, Sang-Bae Ko, MD, PhD, Tae Jung Kim, MD, Jihoon Kang, MD, Hyeon-Min Bae, PhD, Ji Eun Lee, CRC, RN

Supplementary Figure 1. The locations of the channels of NIRSIT

NIRSIT uses 204 channels attached to the forehead of the patient for measurements. Four by 51 channels are located in the frontotemporal area of a brain.

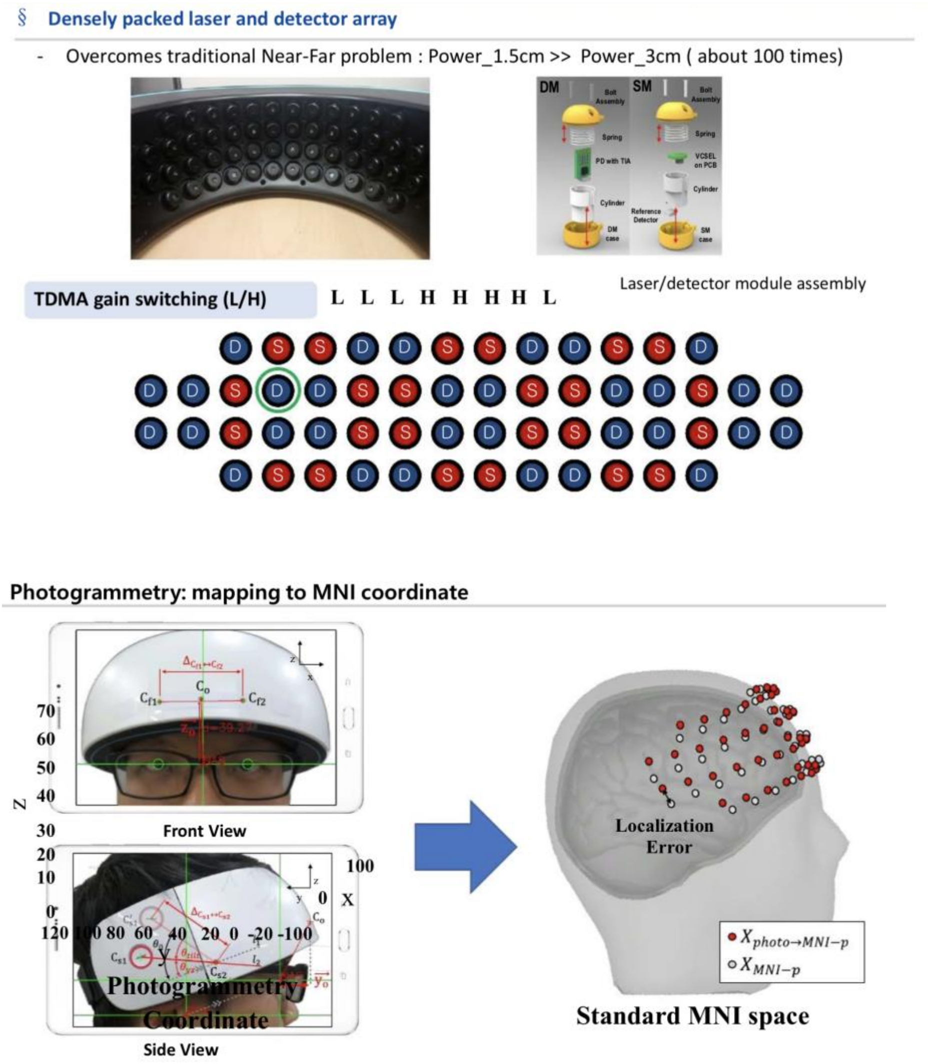

Supplementary Figure 2. The principles in NIRSIT

NIRSIT uses a continuous wave to obtain a regional hemodynamic response from the prefrontal lobe. This device weighs 450 g, making it light and suitable for use as a portable device. NIRSIT has two wavelengths, the 780 nm wavelength that is absorbed by HbR and the 850 nm wavelength that is equally absorbed by HbO2 and HbR. rSO2 was calculated as the concentration percentage HbO2/(HbO2+HbR)×100.

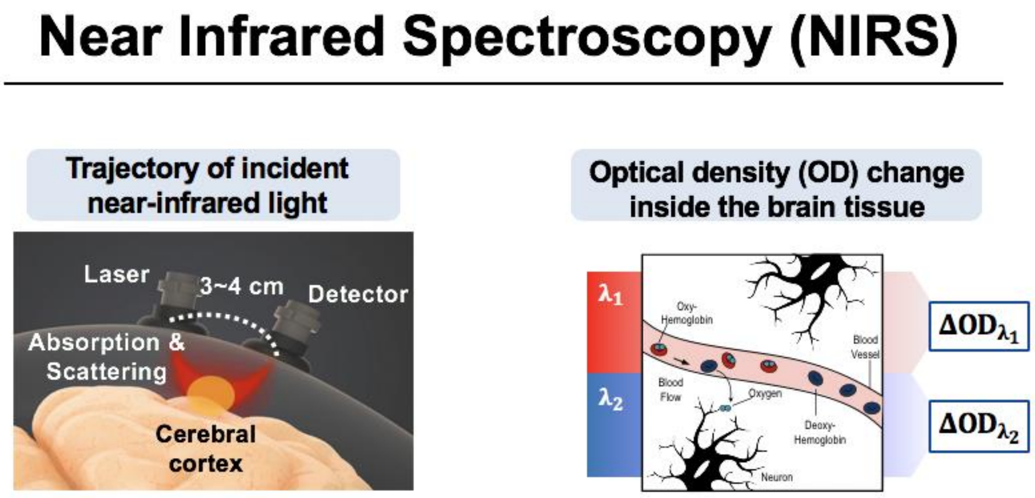

Supplement: Supplementary file 1 [file Data_Sheet_1.PDF]
